# The yeast 14-3-3 proteins BMH1 and BMH2 differentially regulate rapamycin-mediated transcription

**DOI:** 10.1042/BSR20130096

**Published:** 2014-03-18

**Authors:** Michael A. Trembley, Hunter L. Berrus, Jonathan R. Whicher, Emily L. Humphrey-Dixon

**Affiliations:** *St. Lawrence University, 23 Romoda Drive, Canton, NY 13617, U.S.A.

**Keywords:** 14-3-3, Bmh2, nitrogen catabolite repression (NCR), rapamycin, ribosome biogenesis, target of rapamycin (TOR), ChIP, chromatin immunoprecipitation, GO, gene ontology, NCR, nitrogen catabolite repression, qPCR, quantitative PCR, TOR, target of rapamycin, WCE, whole-cell extract, WT, wild-type

## Abstract

14-3-3 proteins are highly conserved and have been found in all eukaryotic organisms investigated. They are involved in many varied cellular processes, and interact with hundreds of other proteins. Among many other roles in cells, yeast 14-3-3 proteins have been implicated in rapamycin-mediated cell signalling. We determined the transcription profiles of *bmh1* and *bmh2* yeast after treatment with rapamycin. We found that, under these conditions, *BMH1* and *BMH2* are required for rapamycin-induced regulation of distinct, but overlapping sets of genes. Both Bmh1 and Bmh2 associate with the promoters of at least some of these genes. *BMH2*, but not *BMH1*, attenuates the repression of genes involved in some functions required for ribosome biogenesis. *BMH2* also attenuates the activation of genes sensitive to nitrogen catabolite repression.

## Abstract 2

Two yeast 14-3-3 proteins, were assumed to have redundant functions because they have nearly identical sequences. Here, we provide evidence that they differentially regulate the transcription of genes in response to inhibition of the TOR pathway by rapamycin treatment.

## INTRODUCTION

14-3-3 proteins are highly conserved and have been found in all eukaryotes investigated. They are involved in many varied cellular processes, and interact with hundreds of other proteins (reviewed in [1]). In yeast, there are two 14-3-3 proteins, Bmh1 and Bmh2, which share 93% amino acid identity [2–4]. In all genetic backgrounds tested, with the exception of Σ1248, deletion of both *BMH1* and *BMH2* is lethal; however, deleting only one of these proteins has little effect on cell growth or viability [4,5]. They most commonly form homodimers or heterodimers, but they have also been reported to act without forming dimers [6]. They function primarily by binding to phosphorylated proteins [7], although other modes of binding have been reported [8–11].

Rapamycin is a small molecule that forms a complex with Fpr1 and the Tor (target of rapamycin) proteins, and thus blocks the TOR signalling pathway [12]. Treating yeast with rapamycin leads to a rapid and robust starvation response including changes in the expression of many yeast genes [13]. Among many other roles in cells, yeast 14-3-3 proteins have been implicated in rapamycin-mediated cell signalling. Bertram et al. found that deleting the yeast 14-3-3 proteins makes cells more sensitive to rapamycin, wherease overexpression of *BMH1* and *BMH2* suppresses the inhibitory effects of rapamycin [14]. In addition, *BMH2* has been shown to be involved in rapamycin-mediated signalling by binding to the transcription factors Msn2/Msn4 and Rtg3 and sequestering them in the cytoplasm. Upon treatment with rapamycin, the transcription factors are released and enter the nucleus [15,16].

The BMH proteins have been shown to localize to chromatin in yeast through several mechanisms including binding to histone H3 that is phosphorylated on serine 10 and acetylated on lysine 14 [17], binding to cruciform DNA [18], and associating with histone acetyltransferases and deacetylases [19]. 14-3-3 proteins have also been found to associate with G-box DNA-binding complexes in several plant species [20–22] and to regulate transcription in both mice and humans [10,23]. The apparent conditional association of 14-3-3 proteins with DNA positions the yeast 14-3-3 proteins as good candidates to regulate gene transcription under multiple growth conditions.

Previous studies have examined the transcription profile of yeast missing both of the BMH proteins under standard growth conditions [24,25]. These studies found that the BMH proteins are mainly required for the transcriptional regulation of genes involved in carbohydrate, lipid, and amino acid metabolism, the stress response, and protein synthesis and folding. They also regulate transporters of amino acids and Rtg3-regulated genes [24,25].

We determined the transcription profiles of *bmh1* and *bmh2* yeast after treatment with rapamycin. We found that, under these conditions, *BMH1* and *BMH2* are required for rapamycin-induced regulation of distinct, but overlapping sets of genes including those involved in ribosome biogenesis and nitrogen catabolite repression (NCR). We show that both Bmh1 and Bmh2 associate with the promoters of at least some of these genes

## MATERIALS AND METHODS

### Yeast strains

The mutant strains *bmh1* (*MAT*a *his3Δ1 leu2Δ0 met15Δ0 ura3Δ0 bmh1::KanMX*) and *bmh2* (*MAT*a *his3Δ1 leu2Δ0 met15Δ0 ura3Δ0 bmh2::KanMX*) and the otherwise isogenic WT (wild-type) strain BY4741 (*MAT*a *his3Δ1 leu2Δ0 met15Δ0 ura3Δ0*) were obtained from Research Genetics. Bmh1-3xHA (MATa BMH1-HA3::URA3::bmh1) and Bmh2–3xHA (MATa BMH2-HA3::URA3::bmh2) were a generous gift from M. P. Longhese (Università di Milano-Bicocca, Milano, Italy).

### Transcription profiling

100 ml yeast were grown to an OD600 of ~1 in yeast extract/peptone/dextrose media and treated with either 50 nM rapamycin or vehicle control [90% (v/v) ethanol, 10% (v/v) Tween-20] for 30 min. Approximately 50 mg of total RNA isolated from these cells was fluorescently labelled and hybridized to DNA microarrays spotted with 70 mer oligos representing all of the known reading frames in the yeast genome as previously described [26]. Microarrays were obtained from Genome Consortium for Active Teaching. These data represent an average of two independent experiments. *K*-means clustering was performed using the program Cluster and visualized in Treeview [27]. Genes that were not up- or down-regulated at least 2-fold in at least one of the cell types were excluded from this analysis.

### RT-qPCR (quantitative PCR)

The coding regions of selected genes were each amplified from 100 ng of total RNA using Qiagen SYBR Green RT-PCR mix in an Opticon 2 real-time PCR machine (Bio-Rad) according to the manufacturers’ instructions. The following primer pairs were used for quantitative RT-PCR: *NSA2*, 5′-CCAAGT-GCGTTGAGACAGAA-3′ and 5′-GTTCGTTTGTGACCTGA-GCA-3′; *NMD3*, 5′-AATGGAACGTGCAGAAAAGG-3′ and 5′-ATTCAACGGGTGTGTTCTCG-3′; *NOG2*, 5′-GGAAACA-CCACACAAGCATTT-3′ and 5′-TTTGGATAGCCGATAAAC-CCTA-3′; *GAP1*, 5′-CCAAAGTATAGAGA-3′ and 5′-TT-GAATTTAGCACC-3′; *BMH1*, 5′- TTTTGACGACGCTATTG-CTG-3′ and 5′- ACTTTGGTGCTTCACCTTCG-3′; *BMH2*, 5′- TCCTGATAAGGCTTGCCACT-3′ and 5′- GCTGT-TGCTGTTGCTGTTGT-3′; *TUB2*, 5′-TCCGGTATGGGTAC-3′ and 5′-ACGTGGGCATTGTAT-3′. Fold-change in expression of each gene was determined using the Pfaffl method [28]. For each gene examined, the coding region of *TUB2* was used as the reference. Each fold-change in expression represents the average of 3–5 independent experiments, each done in duplicate.

### Chromatin immunoprecipitation

DNA associated with Bmh1 or Bmh2 was immunoprecipitated from 90 ml of yeast cells containing endogenously HA-tagged *BMH1* or *BMH2* grown to an OD600 of ~1.0, then treated for 30 min with either 50 nM rapamycin or a vehicle control. Cells were crosslinked and lysed as described [29]. The immunoprecipitation was carried out using 15 μl of anti-HA antibody (Millipore) as described [29]. Approximately 1/50 of the pre-immunoprecipitation material, referred to as WCE (whole-cell extract) was reserved and treated exactly as the immunoprecipitated DNA.

### Quantitative PCR

The promoters of selected genes were each amplified from approximately 1/30 of the immunoprecipitated material using Qiagen SYBR Green PCR mix in an Opticon 2 real-time PCR machine (Bio-Rad) according to the manufacturers’ instructions. The following primer pairs were used for quantitative PCR: *NSA2* promoter, 5′-CAAGGATTCTGATGTCGCAGT-3′ and 5′-GTGCATCTCATCGCTGTCC-3′; *NMD3* promoter, 5′-GGGTGAGAAAATCGCTGTAAA-3′ and 5′-TAGTGTTATG-TACCAGGCGACAA-3′; *NOG2* promoter, 5′-GTGCCAAT-GCTCCCTCTG-3′ and 5′-CGCTTCTTTATATGCCCAAAA-3′; *GAP1* promoter, 5′-AAAGGAGAATAGGG-3′ and 5′-GA-GGTCAATGGGTA-3′; *TUB2* promoter, 5′-GGCCTAA-CAGTAAA-3′ and 5′-GTTGTAGTAGCTGC-3′. Fold binding of Bmh1 and Bmh2 to gene promoters was determined using the Pfaffl method [28]. For each promoter examined, the promoter of TUB2 was used as the reference. Measurements compare the amounts of Bmh1 and Bmh2 recovered in the immunoprecipitated material to amounts recovered in the WCE. Each fold-binding represents the average of 3-5 independent experiments, each done in duplicate.

## RESULTS

### *BMH1* and *BMH2* are required for the rapamycin-induced regulation of different sets of genes

**Figure 1:**
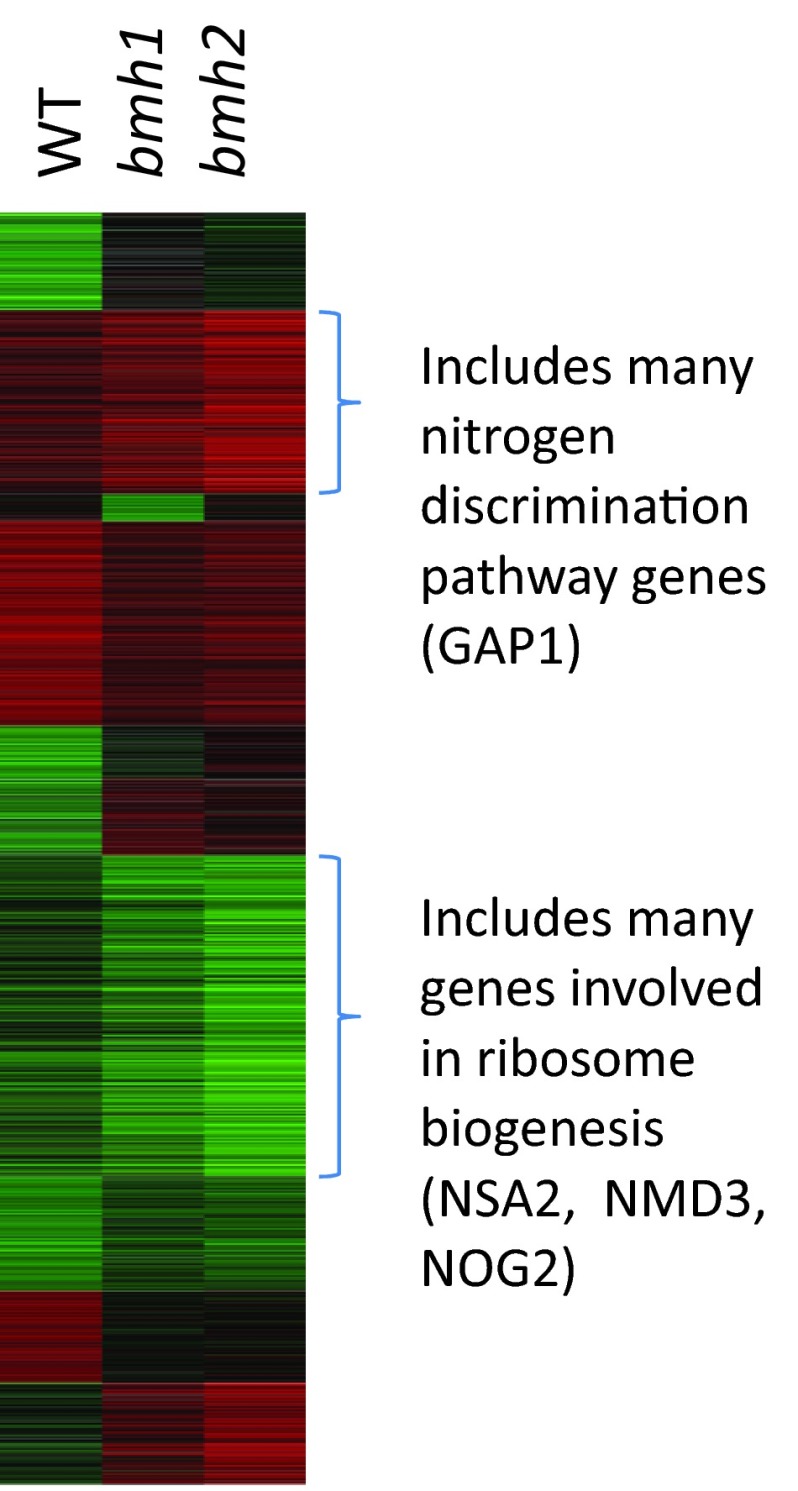
*BMH1* and *BMH2* regulate overlapping gene sets following treatment with rapamycin Log-transformed, averaged transcription profile data sets were clustered using *k*-means with the program Cluster [27]. The results are displayed with Treeview [27]. Red bars indicate genes that were transcriptionally induced, and green bars indicate genes that were transcriptionally repressed. The data set for each strain compares yeast treated with vehicle to those treated with rapamycin.

To determine the roles of *BMH1* and *BMH2* in transcriptional regulation following rapamycin treatment, we used microarrays to examine relative levels of each yeast mRNA. We compared the RNA from yeast treated with rapamycin to those treated with a vehicle control for WT yeast and otherwise isogenic yeast missing the *BMH1* or *BMH2* gene. We obtained similar data from our WT microarray experiment to that obtained in previous studies [13,26], suggesting that these data are reliable. Because the amino acid sequences of *BMH1* and *BMH2* are 93% identical, we expected them to play similar roles in regulating transcription. Indeed, we found that deleting either *BMH1* or *BMH2* alters the rapamycin-induced transcription profile in similar ways, and they are required for both the activation and repression of genes (Figure 1, Supplementary Table S1 at http://www.bioscirep.org/bsr/034/bsr034e099add.htm). However, we also found that *BMH1* and *BMH2* activate and repress distinct sets of genes (Figure 1). Of the top 200 genes that are activated by *BMH1* and *BMH2*, approximately 54% of them overlap.

### *BMH1* and *BMH2* attenuate the repression of different classes of genes

**Figure 2:**
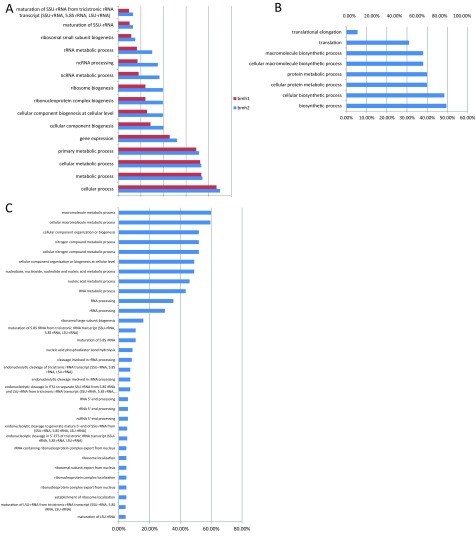
Gene ontology terms enriched (*P*<10^−4^) in the top 200 genes that are transcribed at lower levels in *bmh1* cells or *bmh2* cells than in WT cells following rapamycin treatment were determined (**A**) Enriched gene ontology terms in both *bmh1* and *bmh2* cells, (**B**) enriched gene ontology terms in only *bmh1* cells and (**C**) enriched gene ontology terms in *bmh2*. The per cent of the 200 genes that map to each term is represented in each gene ontology bar.

To determine which classes of genes require *BMH1* and *BMH2* for rapamycin-induced transcriptional regulation, we looked for Gene Ontology (GO) function terms that are associated with the top 200 genes that are either activated or repressed in *bmh1* mutants or *bmh2* mutants more than in WT cells. While genes that perform many functions are regulated by both *BMH1* and *BMH2* following rapamycin treatment (Figure 2A), there are also GO terms that are associated with only the *bmh1* mutant and others that are associated with only the *bmh2* mutant. We found that genes involved in translation are repressed more in a *bmh1* mutant than in WT cells or in a *bmh2* mutant (Figure 2B). In contrast, genes involved in the various steps of ribosome biogenesis are repressed more in a *bmh2* mutant than in WT cells or in a *bmh1* mutant (Figure 2C). We did not find any significant GO terms for genes that are activated more in a *bmh1* or *bmh2* mutant than in WT cells, suggesting that the 14-3-3 proteins act primarily as transcriptional activators following rapamycin treatment. Because there appear to be more genes that require *BMH2* but not *BMH1* for normal levels of transcriptional regulation following rapamycin treatment, and because Bmh1 is present at much higher levels in cells than Bmh2 [30], we focused on genes that depend on BMH2 for regulation.

### *BMH2* is required to attenuate the repression of ribosome biogenesis genes

**Figure 3:**
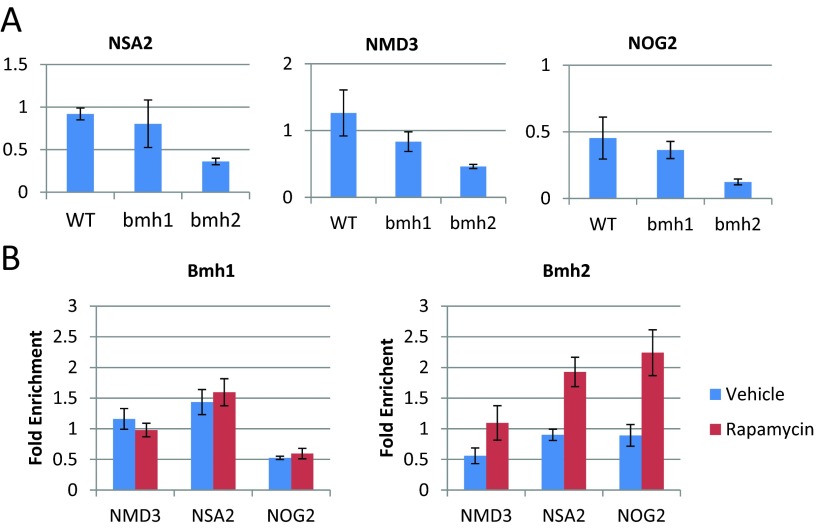
*BMH2* but not *BMH1* acts at the promoters of ribosome biogenesis genes to attenuate their repression following rapamycin treatment (**A**) Quantitative RT-PCR was used to determine the transcriptional effect of treating the indicated yeast strain (WT, *bmh1* and *bmh2*) with rapamycin. Each data point represents the abundance of *NMD3*, *NSA2* and *NOG2* RNA in rapamycin-treated cells compared with vehicle-treated cells, relative to the RNA levels of TUB2. Error bars indicate the standard error of the mean (*n*=3). (**B**) ChIP followed by quantitative PCR was used to determine the extent to which Bmh1 and Bmh2 bind to the promoters of *NMD3*, *NSA2* and *NOG2*. Each data point represents the fold enrichment of the indicated promoter after the immunoprecipitation compared with the whole cell extract, relative to the enrichment of the promoter of *TUB2.* Error bars indicate the standard error of the mean (*n*=4).

Treating yeast with rapamycin leads to a decrease in translation, which is caused in part by transcriptional down-regulation of genes involved in ribosome biogenesis [13]. Based on the microarray data, *BMH2* but not *BMH1* acts to decrease the repression of many ribosome biogenesis genes. We selected three ribosome biogenesis genes, *NSA2*, *NMD3* and *NOG2*, and used quantitative reverse-transcription PCR (RT-qPCR) to verify this observed decrease in repression (Figure 3A). We found that Bmh2, but not Bmh1, attenuates the repression of all of these genes following rapamycin treatment. Next, we wanted to know whether the transcriptional regulation of these genes by *BMH2* was direct or indirect. To do this, we used ChIP (chromatin immunoprecipitation) followed by qPCR. We found that Bmh2 associates with the promoters of these genes following rapamycin treatment (Figure 3B), suggesting that this is a direct effect.

### *BMH2* is required to attenuate the activation of NCR genes

**Figure 4:**
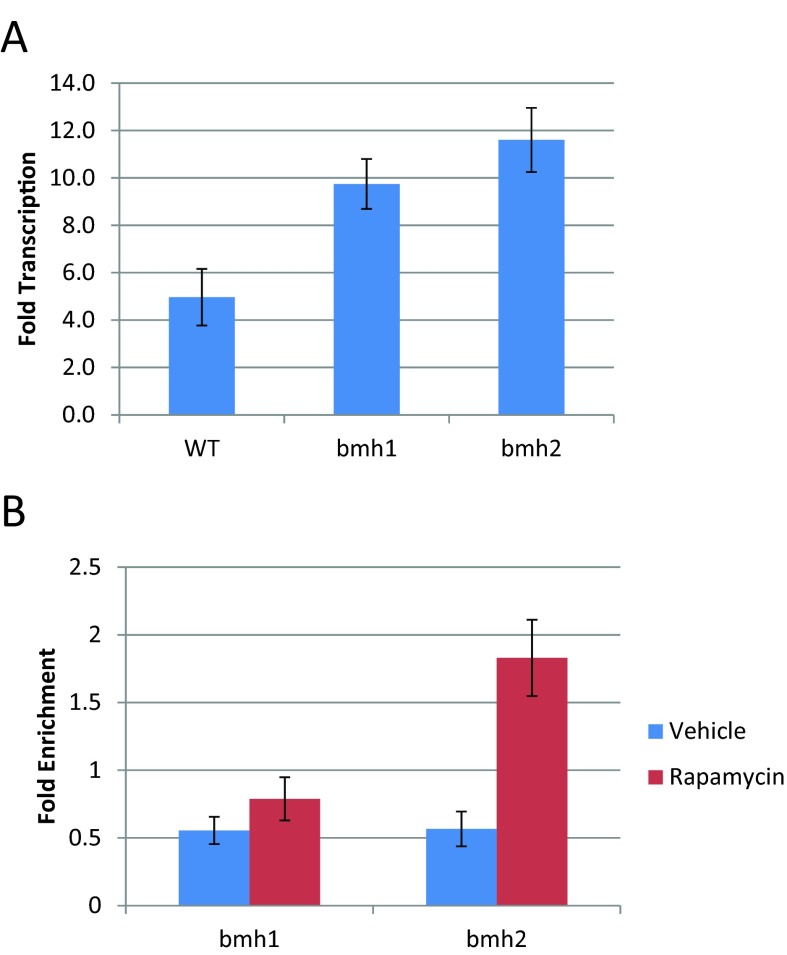
*BMH2* but not *BMH1* acts at the promoter of *GAP1* to decrease its activation following rapamycin treatment (**A**) Quantitative RT-PCR was used to determine the transcriptional effect of treating the indicated yeast strain (WT, *bmh1* and *bmh2*) with rapamycin. Each data point represents the abundance of *GAP1* RNA in rapamycin-treated cells compared with vehicle-treated cells, relative to the RNA levels of *TUB2*. Error bars indicate the standard error of the mean (*n*=3). (**B**) ChIP followed by quantitative PCR was used to determine the extent to which Bmh1 and Bmh2 bind to the promoter of *GAP1*. Each data point represents the fold enrichment of the promoter of *GAP1* after the immunoprecipitation compared with the whole cell extract, relative to the enrichment of the promoter of *TUB2.* Error bars indicate the standard error of the mean (*n*=4). Based on the microarray data, *GAP1* is activated upon rapamycin treatment and deleting *BMH1* or *BMH2* increases that activation.

The microarray data indicate that genes sensitive to NCR are more highly expressed in *bmh2* mutants than in *bmh1* mutants. These genes are activated to approximately the same extent in WT and *bmh1* cells, but activated, on average, approximately 1.5-fold more in *bmh2* mutants than in WT cells or *bmh1* mutants. We examined the transcription of *GAP1*, an NCR gene, by quantitative RT-PCR to verify these microarray results. We found that *BMH2* attenuates the activation of *GAP1* to a greater extent than does *BMH1* (Figure 4A), verifying our microarray data. Bmh2 binds to the promoter of *GAP1* upon rapamycin treatment (figure 4B), suggesting that this is a direct effect.

### The yeast 14-3-3 proteins do not regulate each other's expression

**Figure 5:**
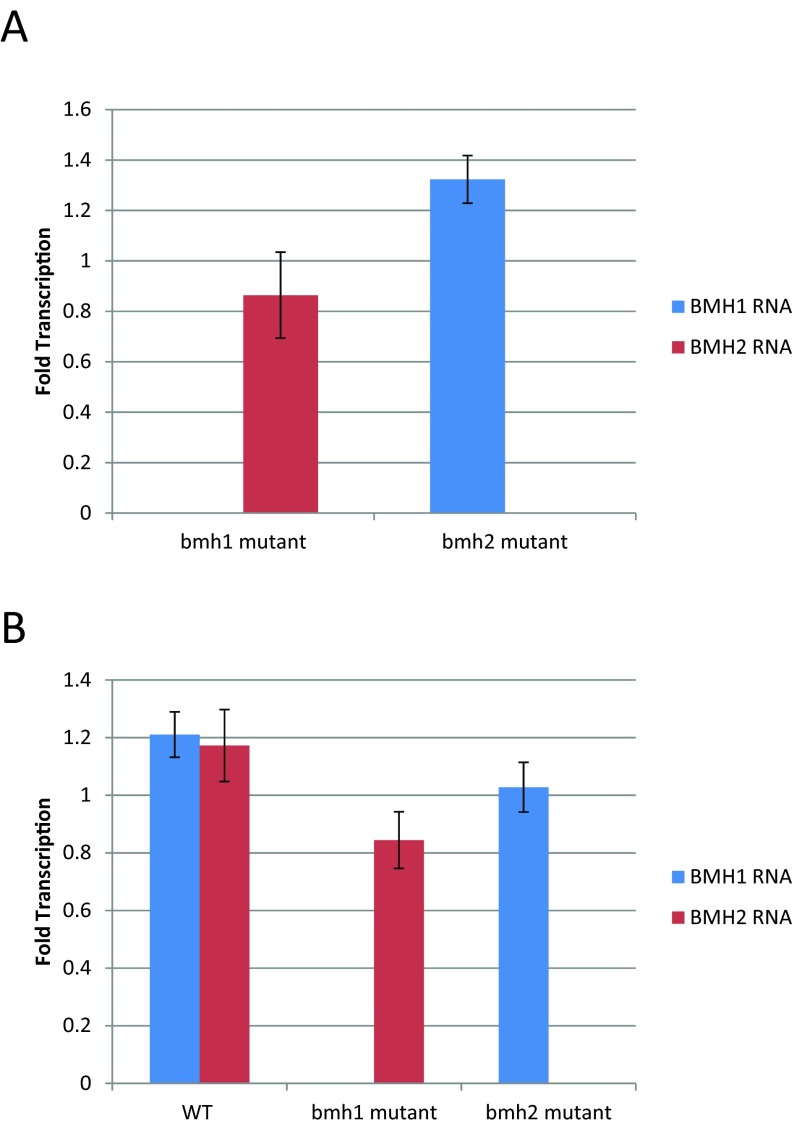
Deleting one 14-3-3 gene does not alter the expression of the other 14-3-3 gene Quantitative RT-PCR was used to determine the transcriptional effect of deleting one 14-3-3 gene in (**A**) untreated cells and (**B**) cells treated with rapamycin. Each data point represents the abundance of BMH1 or BMH2 RNA in the indicated mutant cells compared with WT cells (**A**) or in rapamycin-treated cells compared with vehicle-treated cells, relative to the levels of TUB2. Error bars indicate the standard error of the mean (*n*=3).

One possible explanation for our data is that the absence of one of the yeast 14-3-3 genes could lead to altered expression levels of the other 14-3-3 gene. To test this possibility, we examined the expression of *BMH1* and *BMH2* when the other 14-3-3 gene was deleted both in untreated cells and in cells treated with rapamycin. We found that deleting one 14-3-3 gene does not lead to changes in the expression of the other 14-3-3 gene (Figure 5).

## DISCUSSION

Here, we present evidence that *BMH1* and *BMH2* play overlapping but distinct roles in regulating rapamycin-induced transcriptional changes. Previous genome-wide studies of the effects of yeast 14-3-3 proteins on transcription used systems in which both *BMH1* and *BMH2* were inactivated, and looked only at steady-state conditions [24,25]. We found some of the same gene classes to be regulated by individual 14-3-3 proteins following rapamycin treatment, but also identified new groups of genes that require 14-3-3 proteins for transcriptional regulation following rapamycin treatment.

To the best of our knowledge, this is the first report of Bmh1 and Bmh2 performing different roles in regulating transcription. However, Bmh1 and Bmh2 appear to be required for some different processes in cells. For example, previous results demonstrate that Bmh1, but not Bmh2 is required for the efficient forward transport of Pmp2 [31] and that the two yeast 14-3-3 proteins have different subcellular localization patterns [32]. This suggests that while Bmh1 and Bmh2 have very similar sequences, there is sufficient variability to allow them to perform unique functions.

We identified genes that are regulated by just Bmh1, just Bmh2, or both Bmh1 and Bmh2 following rapamycin treatment. Genes that require one of the yeast 14-3-3 proteins but not the other for WT expression levels likely have a requirement for the corresponding 14-3-3 protein homodimer. Genes that have altered expression in both *bmh1* and *bmh2* mutants likely require a Bmh1/Bmh2 heterodimer for proper expression, or require a 14-3-3 protein to be present and are sensitive to changes in the overall levels of 14-3-3 proteins caused by deleting one of the isoforms. All three of these dimer types occur *in vivo*, with approximately 65% of Bmh2 and 79% of Bmh1 occurring in homodimers [33]. However, since there are approximately 3.3 copies of Bmh1 for every copy of Bmh2, all three dimer types are likely to be present in sufficient abundance to allow for functionality [30,33].

Our data suggest that there is a stronger role for Bmh2 than Bmh1 in regulating rapamycin-mediated transcription. This could be explained if there were higher levels of Bmh2 than Bmh1. In fact, the opposite result has been found [30]. This suggests that our results are not due to a dosage effect. We also show that our results are not a due to deleting one of the 14-3-3 isoforms altering the expression levels of the other isoform. In order to control transcription, however, proteins must be localized to the nucleus. Under steady-state conditions, Bmh2 is localized to both the cytoplasm and the nucleus, whereas Bmh1 is localized only to the cytoplasm [32]. Since we have identified a role for Bmh1 in regulating transcription, it is likely that there is at least a small amount of Bmh1 in the nucleus following rapamycin treatment. Bmh1 becomes localized to the nucleus upon DNA replication stress [34], suggesting that such a translocation is possible.

Our data suggest that Bmh2 is necessary for the regulation of gene classes including ribosome biogenesis genes and NCR genes. Bmh2 binding to the promoters of these genes increases following rapamycin treatment, suggesting that Bmh2 is directly regulating these genes. This regulation may be due to Bmh2 binding to either the DNA or other proteins in the promoters of these genes.

We found that both Bmh1 and Bmh2 act to moderate many of the transcriptional effects of rapamycin treatment, including the down-regulation of ribosome biogenesis genes and the up-regulation of NCR genes. This is consistent with Bertram et al. [14] who showed that the 14-3-3 proteins decrease rapamycin-induced growth suppression, and also with Wang et al. [35] who showed that deleting *BMH1* extends lifespan by enhancing the stress response, and does so in part through interactions with the TOR pathway.

We found that Bmh2 attenuates the activation of NCR-sensitive genes. Following rapamycin treatment, the transcription factors Gln3 and Gat1 translocate into the nucleus and activate the transcription of these genes. It was previously determined that the yeast 14-3-3 proteins bind Gln3 and Gat1 [36], suggesting a potential mechanism for this effect.
